# Chitosan-Based Glycolipid Conjugated siRNA Delivery System for Improving Radiosensitivity of Laryngocarcinoma

**DOI:** 10.3390/polym13172929

**Published:** 2021-08-30

**Authors:** Jing Miao, Liwen Zhang, Peng Gao, Huawei Zhao, Xianji Xie, Junyan Wang

**Affiliations:** 1 Department of Pharmacy, The Children’s Hospital, Zhejiang University School of Medicine, National Clinical Research Center for Child Health, Hangzhou 310052, China; miaojing@zju.edu.cn (J.M.); 6512070@zju.edu.cn (L.Z.); gaopeng@zju.edu.cn (P.G.); zhaohuawei@zju.edu.cn (H.Z.); 2 Department of Pharmacy, The First Affiliated Hospital, Zhejiang University School of Medicine, Hangzhou 310003, China

**Keywords:** GLUT-1, siRNA, chitosan oligosaccharide grafted stearic acid, laryngocarcinoma, radiosensitization

## Abstract

Glucose Transporter-1 (GLUT-1) is considered to be a possible intrinsic marker of hypoxia in malignant tumors, which is an important factor in radioresistance of laryngocarcinoma. We speculated that the inhibition of GLUT-1 expression might improve the radiosensitivity of laryngocarcinoma. GLUT-1 siRNA was designed to inhibit the GLUT-1 expression, but the high molecular weight and difficult drug delivery limited the application. Herein, we constructed a glycolipid polymer chitosan oligosaccharide grafted stearic acid (CSSA) to conjugate siRNA via electrostatic interaction. The characteristics of CSSA and CSSA/siRNA were studied, as well as the radiosensitization effect of siRNA on human laryngocarcinoma epithelial (Hep-2) cells. Compared with the traditional commercial vector Lipofectamine^TM^2000 (Lipo), CSSA exhibited lower cytotoxicity, more efficiently cellular uptake. Incubating with CSSA/siRNA, the survival rates of Hep-2 cells were significantly decreased comparing with either the group before transfection or Lipo/siRNA. CSSA is a promising carrier for efficient siRNA delivery and radiosensitization of laryngocarcinoma.

## 1. Introduction

Laryngocarcinoma is a common malignant tumor in the head and neck. Although early laryngocarcinoma has been actively treated by surgery or radiotherapy, the five-year local recurrence rate is still 5–10%. Among the laryngocarcinoma patients receiving radiotherapy, 5–10% of clinical stage I patients and up to 25% of clinical stage II patients have resistance to radiotherapy, which affects the curative effect of radiotherapy [1]. Radiotherapy plays an important role in the comprehensive treatment of laryngocarcinoma, but laryngocarcinoma has low sensitivity to radiotherapy, strong resistance, treatment resistance and recurrence has always been a difficult and unsolved problem in the treatment of laryngocarcinoma. Therefore, it is necessary to explore effective radiosensitizers, reduce radiation side effects, improve the cure rate of laryngocarcinoma, and improve the survival time and quality of life.

There are many factors affecting the radiation effect of tumor, among which the hypoxia and reoxygenation of tumor tissue play an important role. The radiotherapy dosage for the tumor cells with severe hypoxia is 2–3 times higher than for those with sufficient oxygen supply [2,3,4,5]. Therefore, the existence of hypoxia cells in malignant tumor is one of the important reasons for radioresistance. Tumors grow rapidly with significant energy requirement, and the glycolysis under hypoxia situation consumes more glucose than the glucose oxidation, thus the glucose metabolic rates of tumor cells are higher than that of normal cells. In addition, compared with normal cells, tumor cells still use glycolysis as the main form of production capacity even under the condition of sufficient oxygen supply, namely “Warburg effect” [6,7]. As a result, the rapid growth of malignant tumor depends on a large amount of glucose intake. 

Glucose Transporter (GLUT) is a transmembrane glycoprotein on the cell membrane, which mediates the transport of glucose inside and outside the cells in the form of facilitated diffusion. There are 13 kinds of glucose transporters, including GLUT 1–12 and Hα-inositol receptors are encoded by SLC2A1–13 gene [8,9]. Among them, GLUT-1, widely distributed in human tissues, has the highest correlation with malignant tumors and plays a leading role in glucose absorption and transportation [10,11,12,13,14,15]. GLUT-1 is a rate limited transporter, and its increased glucose transport depends on the increased expression of GLUT-1 protein [16].

GLUT-1 is expected to be a new tumor marker for the prognosis of malignant tumors. Many studies have shown that the expression of GLUT-1 cannot be detected by polyclonal antibody immunohistochemistry in human normal and benign tumor tissues, but it is generally highly expressed in malignant tumor tissues [17,18,19]. The physiological environment of malignant tumor cells is relatively lack of energy. The overexpression of GLUT-1 can transport more glucose to meet the needs of high metabolic rate and rapid growth of malignant tumor cells, promote the absorption and utilization of glucose by malignant tumor cells, accelerate glycolysis, improve the survival ability of tumor cells, and accelerate the tumor growth. As the poor prognosis is closely related to the tolerance to chemoradiotherapy, the overexpression of GLUT-1 plays an important role in the survival of malignant tumors.

As reported by some previous studies [20,21], there is also high expression of GLUT-1 in laryngocarcinoma, and the expression of GLUT-1 is related to the radioresistance of laryngocarcinoma. Thus, inhibiting the expression of GLUT-1 can improve the radiosensitivity of laryngocarcinoma. 

Although the abnormal expression of GLUT-1 is not the only explanation for tumor radioresistance, its significance in tumor radiobiology has been paid more and more attention. Inhibiting the expression of GLUT-1 to improve the radiosensitivity has become one of the hotspots in targeted therapy for malignant tumors. Herein, we synthesize the GLUT-1 siRNA (siRNA803) to inhibit the expression of GLUT-1 in human laryngocarcinoma epithelial (Hep-2) cells according to the previous studies [22,23]. However, siRNA is a hydrophilic macromolecular substance with negative charge, and difficult to penetrate the cell membrane by itself, so it needs to be transfected with effective vectors. Additionally, siRNA is easy to be degraded by various enzymes in blood and liver, and it is also easy to be degraded by enzymes in lysosomes even after it enters cells with vector. Therefore, the genetic drugs need to be effectively protected during the drug delivery process, and further can be rapidly released from lysosomes after entering cells. For genetic drugs, viral vectors have the characteristics of high efficiency of cell transfection, but there are safety problems such as immune response induced by viral protein and potential virus replication in vivo [24]. Cationic liposomes are mostly used as the non-viral vectors for siRNA, but the application was restricted by the high cytotoxicity and the low gene delivery efficiency [25]. Our previous studies [26,27] have demonstrated that chitosan oligosaccharide grafted stearic acid (CSSA), which is synthesized by chemical grafting of carboxyl group of stearic acid and active amino group of chitosan oligosaccharide, can self-assemble into micelles in the aqueous medium and tightly bind negative-charged genetic drugs through the electrostatic interaction. The gene delivery micelles composed of CSSA have good biological stability and safety. CSSA both has the merits of chitosan, such as biocompatibility, biodegradability, low cytotoxicity in comparison to other cationic polymers [28,29], and also has the merits of stearic acid, which could be rapidly internalized into carcinoma cells [30,31]. Moreover, by controlling the degree of amino substitution of CSSA, cytoplasmic residence [26,27] or nuclear tropism [32,33] can be realized. All the above suggest that CSSA could be an efficient vector for siRNA delivery.

In this research, we synthesized siRNA803, a kind of GLUT-1 siRNA which could efficiently inhibit the expression of GLUT-1. The CSSA micelles were constructed and conjugate siRNA by electrostatic interaction to form CSSA/siRNA, which could be an efficient approach for intracellular delivery of siRNA. The characteristics of CSSA and CSSA/siRNA were studied, as well as the radiosensitization effect of GLUT-1 siRNA on Hep-2 cells, investigating the cellular mechanisms underlying the enhancement of laryngocarcinoma radiosensitivity by GLUT-1 siRNA.

## 2. Results and Discussion

### 2.1. Synthesis and Characteristics of CSSA

**Figure 1:**
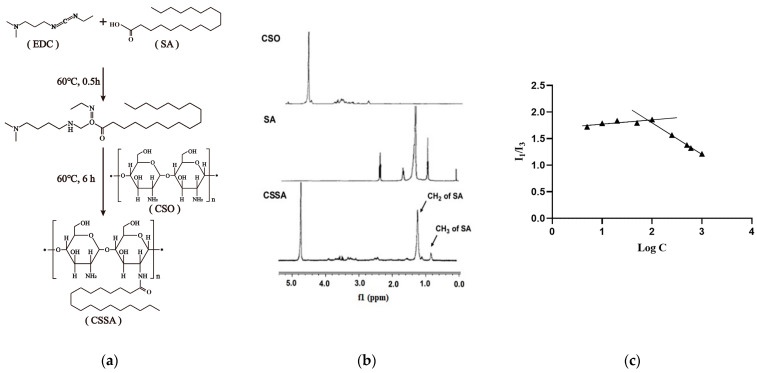
Synthesis and characterizations of CSSA. (**a**) Synthesis route of CSSA; (**b**) ^1^H NMR spectra of CSO, SA and CSSA; (**c**) Variation of intensity ratio (I_1_/I_3_) vs. concentration (μg/mL) of CSSA (▲).

Glycolipid polymer CSSA was constructed as shown in Figure 1a. Low molecular weight chitosan (Chitosan oligosaccharide) obtained by enzymatic hydrolysis was chemically grafted with carboxyl group of stearic acid and active amino group of chitosan oligosaccharide by the EDC method to synthesize CSSA.

The chemical structure of CSSA was confirmed by ^1^H-NMR (Figure 1b). The proton peaks of -CH2 and -CH3 in SA molecules appeared in the ^1^H-NMR spectrum of CSSA, but there were no such peaks at the same position in the ^1^H-NMR spectrum of CSO. These results indicated that a new chemical bond was formed between the carboxyl group of SA and active amino group of CSO, which proving the successful synthesis of the CSSA. 

As shown in Table 1, the substitution degree (SD), defined as the molar ratio of stearate to anhydroglucosidic units in chitosan oligosaccharide, was measured as 15.70 ± 1.02%. The synthesized glycolipid polymers CSSA could self-assemble into micelles with an average diameter of 144.7 ± 3.2 nm and a zeta potential of 39.7 ± 0.8 mV. The critical micelle concentration (CMC) of CSSA was determined by taking a constant amount of pyrene as a fluoroprobe [34] and varying the concentration of CSSA from 1.0 × 10^−3^ to 1.0 mg/mL. An excitation wavelength of 337 nm was used in all instances for the selective excitation of pyrene and the emission spectra were recorded from 360 to 450 nm. As Figure 1c shown, the CMC was evaluated by plotting the ratio of the first peak (I_1_, 374 nm) to the third peak (I_3_, 385 nm) in the pyrene emission spectra against the logarithmic concentration (Log C) of CSSA. The in-flection point corresponds to the CMC value of CSSA was 75.02 ± 1.27 μg/mL (listed in the Table 1). 

### 2.2. Preparation and Characteristics of CSSA/siRNA

**Figure 2:**
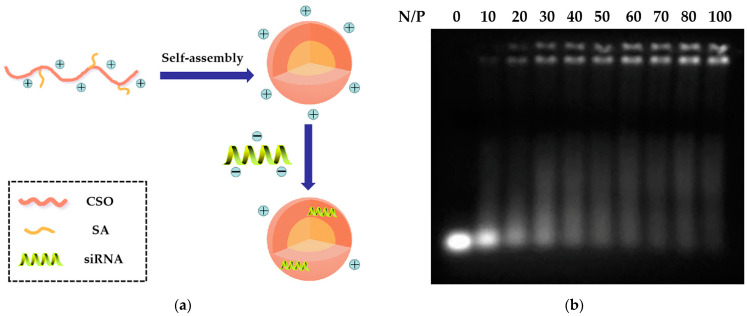
Preparation of CSSA/siRNA. (**a**) The schematic diagram; (**b**) Gel retarding analysis.

As shown in Figure 2a, CSSA was used to construct siRNA delivery systems by electrostatic combination. The siRNA condensation capacity of CSSA micelles was analyzed by a gel retardation assay using an agarose gel electrophoresis. Figure 2b shows the gel retardation result of CSSA/siRNA at different N/P ratios from 0 to 100. When the N/P ratio was above 60, CSSA/siRNA was almost retarded in the sample hole, which suggested CSSA micelles and siRNA started to form tight complexes. Therefore, by gel retardation electrophoresis analysis, CSSA/siRNA were constructed at the optimized N/P of 60 in our following studies. As shown in Table 1, the average diameter of CSSA/siRNA with N/P of 60 was determined as 168.1 ± 2.9 nm, which was larger than that of CSSA, resulting from siRNA adherence on the surface of CSSA via electrostatic interaction. The zeta potential of CSSA/siRNA was determined as 20.3 ± 0.5 mV, which was lower than that of CSSA, resulting from that the negatively charged siRNA neutralized part of the positive charge of CSSA after binding.

### 2.3. Cytotoxicity

**Figure 3:**
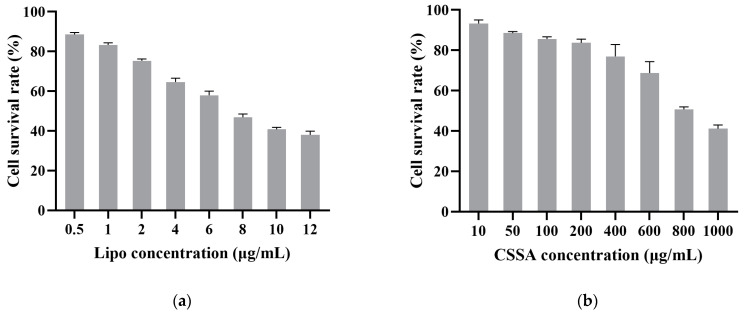
Cytotoxicity of Lipo (**a**) and CSSA (**b**) against Hep-2 cells (n = 3).

A MTT evaluation of the in vitro cytotoxicity of CSSA and Lipo against Hep-2 cells was carried out. As shown in Figure 3, the cellular growth inhibition (IC_50_) of Lipo was measured to be 7.3 μg/mL, while the IC_50_ of CSSA was measured to be 880.5 μg/mL. The results revealed that CSSA possessed much lower cytotoxicity than Lipo, which indicated the CSSA micelles systems much safer carrier for siRNA than traditional Lipo. 

### 2.4. Internalization of FAM-siRNA

**Figure 4:**
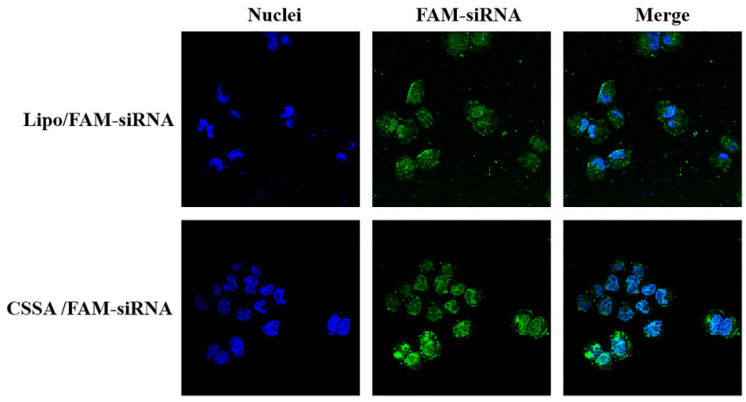
The intracellular localization of FAM-siRNA transfected by Lipo and CSSA after 4 h on Hep-2 cells.

5-Carboxyfluoresce (FAM) is a green fluorescent group with excitation wavelength of 480 nm and emission wavelength of 520 nm. Only when FAM-siRNA was successfully taken up by Hep-2 cells, FAM-siRNA scattered in cytoplasm can be observed. As Figure 4 shown, In the Hep-2 cells incubated with CSSA/FAM-siRNA, a large number of green cells were observed. The fluorescence intensity (A.U.) analyzed by ImageJ of CSSA/FAM-siRNA group was stronger to that of Lipo/FAM-siRNA group (65.363 vs. 84.49, *p* < 0.05), indicating that CSSA could effectively deliver siRNA with better ability than Lipo. On the other hand, most of the nucleus was kept ovoid-shaped after incubation with CSSA, while a number of rotund cells were observed after incubation with Lipo. The form change of cells related with the cytotoxic of material, and also influenced the internalization efficiency. As a result, more FAM-siRNA were uptake by the more viable cells.

### 2.5. Cell Survival Rate with X-ray Radiation

**Figure 5:**
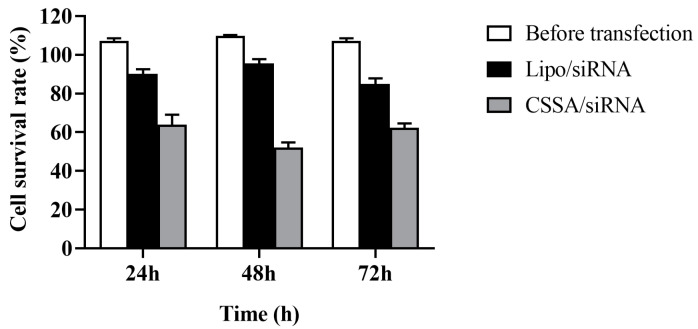
The cell survival rate underwent 5 Gy of X-ray radiation during different culture time (n = 3).

As Figure 5 shown, before transfection (BR), cell survival rates of Hep-2 cells underwent 5 Gy X-ray radiation were no significant difference during different culture time (24 h, 48 h, 72 h) (*p* > 0.05), suggesting that Hep-2 cells were radioresistant to X-ray radiation.

After transfection of GLUT-1 siRNA, cell survival rates of both Lipo/siRNA and CSSA/siRNA groups were gradually reduced with the prolongation of culture time (*p* < 0.05) also underwent 5 Gy X-ray radiation. At the same time, the cell survival rates of Lipo/siRNA group was significantly reduced comparing with BR group (*p* < 0.05), suggesting that transfection could reduce the radiation resistance of Hep-2 cells. Moreover, the cell survival rates of CSSA/siRNA group was also significantly reduced comparing with BR group (*p* < 0.05), further significantly reduced comparing with Lipo/siRNA group (*p* < 0.05). Although both of CSSA and Lipo having cationic and hydrophobic segments, the function of CSSA is better than Lipo, which may be relates with the specific structure [35]. The amphiphilic graft copolymer composed of CSO as a backbone and SA as graft was expected to have a core-shell structure, thus, hydrophobic SA segments as an internal core and cationic CSO as a surrounding corona. Due to the longer main chain of CSO and shorter graft chain of SA, a core-shell structured CSSA micelle with some SA “minor core” near the shell of micelle is considerable, which may favor the escape of CSSA/siRNA from endosome. In addition, CSSA inhibition better ability to deliver siRNA than Lipo, as shown in Figure 4. Consequently, CSSA/siRNA could efficiently increase the sensitivity of radiotherapy, and even better than the traditional commercial vector Lipo.

## 3. Materials and Methods

### 3.1. Materials 

Chitosan oligosaccharide (CSO) of low molecular weight (MW = 18.4 kDa) was obtained by enzymatic degradation of chitosan (95% deacetylated, MW = 450 kDa), and purchased from Yuhuan Marine Biochemistry (Zhejiang, China). Stearic acid (SA) was supplied by Shanghai Chemical Reagent (Shanghai, China). The compounds 2,4,6-trinitrobenzene sulfonic acid (TNBS) and Methylthiazoletetrazolium (MTT) were purchased from Sigma (St. Louis, MO, USA). 1-ethyl-3-(3-dimethylaminopropyl) carbodiimide (EDC) and pyrene were provided by Aladdin (Shanghai, China). Bis Benzimide Hoechst NO33342 (Hoechst33342) was purchased from Asros organics (Belgium, USA). Lipofectamine^TM^2000 (Lipo) and Fetal bovine serum (FBS) were purchased from Invitrogen (Carlsbad, CA, USA). Trypsin and Dulbecco’s minimum essential medium (DMEM) and Fetal bovine serum were purchased from Gibco (Merelbeke, Belgium). Other chemicals used were of analytical or chromatographic grade. 

### 3.2. Cell Culture 

Hep-2 cells were purchased from the Cell Research Institute of the Chinese Academy of Sciences (Shanghai, China) and cultured in DMEM supplemented with 10% FBS, 100 U/mL penicillin, 100 U/mL streptomycin, and 380 µg/mL geneticin G418 in a humidified atmosphere containing 5% CO_2_ at 37 °C. 

### 3.3. Design and Synthesis of siRNA 

siRNA803 for inhibiting the expression of GLUT-1 was designed and consigned synthesis to GenePharma (Shanghai, China). The sequences of siRNA803 were:

sense, 5′-GGAAUUCAAUGCUGAUGAUTT-3′;
antisense, 5′-AUCAUCAGCAUUGAAUUCCTT-3′.

### 3.4. Synthesis of CSSA

Chitosan oligosaccharide (CSO) was prepared by enzymatic degradation of chitosan according to the previous study [36]. The molecular weight of CSO with 18.4 kDa molecular weight used in this paper was determined by gel permeation chromatography (GPC) with TSK-gel column (G3000SW, 7.5 mm i.d. × 30 cm). CSSA was then synthesized via a reaction of the carboxyl group of SA with the amine group of the CSO in the presence of EDC. Briefly, 400 mg CSO was dissolved in 40 mL deionized water, and 100 mg SA and 380 mg EDC were dissolved in 20 mL ethanol by sonicating treatment (Sonic Purger CQ250, Academy of Shanghai Shipping Electric Instrument) for 30 s, respectively. The CSO solution was heated to 60 °C under stirring accompanied by dropwise addition of SA solution. The reaction mixtures were stirred for 6 h and then dialyzed against deionized water using a dialysis membrane (molecular weight cut-off (MWCO): 7 kDa, Spectrum Laboratories, Laguna Hills, CA, USA) for 48 h. Finally, the product was collected by lyophilization followed by a further wash with ethanol to remove products. The washed product was then collected by lyophilization again.

CSO, SA, and CSSA were dissolved in Deuterium oxide (D_2_O), and the chemical structures were characterized by ^1^H NMR with an AVANCE DMX 500 NMR spectrometer (Bruker, Rheinstetten, Germany).

### 3.5. Preparation of CSSA/siRNA Complex and Gel Retardation Assay

CSSA solutions with different concentration (25 mM sodium acetate buffer, pH 5.5) were firstly purified by 0.22 μm millipore filter. The stable CSSA/siRNA complexes with various N/P (The N/P was the ratio of the number of unreacted free primary amines of CSSA and the number of phosphate groups of siRNA) were prepared by mixing the appropriate volume of CSSA micelle solution and siRNA solution (500 μg/mL) vortically for 30 s, and then incubating for 30 min at 37 °C. 

Electrophoresis was then carried out with a current of 100 V for 20 min in TAE buffer solution (40 mM Tris–HCl, 1% (*v*/*v*) acetic acid, 1 mM EDTA). The retardations of the CSSA/siRNA complexes with various N/P ratios were visualized by the staining of ethidium bromide.

### 3.6. Characterization of CSSA and CSSA/siRNA

#### 3.6.1. Size and Zeta Potential 

The size and zeta potential of CSSA micelles and CSSA/siRNA with N/P of 60 were measured by Dynamic Light Scattering (Zetasizer 3000HSA, Malvern Instruments, Worcestershire, UK). Size presented in this section is volume average diameter.

#### 3.6.2. Substitution Degree of Amino Groups 

The substitution degree of amino groups (SD) was determined by the TNBS method [37]. 2 mL of 4% NaHCO_3_ and 2 mL of 0.1% TNBS solution were added into 2 mL of CSSA solution with 125 μg/mL CSSA, and the mixture was incubated at 37 °C for 2 h. 2 mL of HCl (2 N) was then added into the mixture to neutralize the remaining NaHCO_3_. The ultra-violet (UV) absorbance of final reaction mixture at 344 nm was measured by UV spectroscopy (TU-1800PC, Purkinje General Instrument, Beijing, China).

#### 3.6.3. Critical Micelle Concentration 

The critical micelle concentration (CMC) of CSSA was estimated by fluorescence spectroscopy using pyrene as a probe. The excitation wavelength was 337 nm, and the slit was set at 2.5 nm (excitation) and 10 nm (emission). The intensities of the emission were monitored by a fluorometer (F-2500, Hitachi, Tokyo, Japan) at a wavelength range of 360–450 nm. The concentrations of CSSA solution containing 5.93 × 10^−7^ M of pyrene were varied from 1.0 × 10^−3^ to 1.0 mg/mL. The intensity ratio (I_1_/I_3_) of the first peak (I_1_, 374 nm) to the third peak (I_3_, 385 nm) in the pyrene emission spectra was analyzed to calculate CMC. 

### 3.7. Cytotoxicity Assay

Cell cytotoxicity of Lipo and CSSA were determined by MTT assay. Hep-2 cells were seeded at a density of 1.0 × 10^4^ cells/well in 96-well culture plates and incubated for 48 h. Lipo and CSSA were added into 96-well culture plates. Blank culture medium was treated as a control. After incubating for further 72 h, 20 μL of MTT solution (5.0 mg/mL) was added and incubated for another 4 h. Thereafter, the medium was replaced by 200 μL of DMSO to dissolve the purple formazan crystals. The sample absorbance of each well was measured at 570 nm by a microplate reader (Model 680, BIO-RAD, Berkeley, CA, USA). Cell viability was calculated with reference to the cells incubated in the culture medium alone. Dose-effect curves were plotted from the data corresponding to the triplicate assays. 

### 3.8. Internalization of CSSA Micelles

5-Carboxyfluorescein (FAM) was used to label siRNA, and CSSA/FAM-siRNA with N/P of 60 was prepared as described in Section 3.5. Lipo/FAM-siRNA was performed as positive controls according to the manufacture’s protocol.

Hep-2 cells were seeded at a density of 2.0 × 10^3^ cells/well in 24-well culture plates and incubated for 48 h. Thereafter, cells were incubated with Lipo/FAM-siRNA or CSSA/FAM-siRNA (the Lipo and CSSA concentration were 100 μg/mL) for 4 h and then were washed thrice with PBS (pH 7.4). The nucleus was stained with Hoechst33342, and the cellular uptakes of FAM-siRNA delivery by different vectors were observed under a confocal laser scanning microscopy (CLSM, Ix81-FV1000, Olympus, Tokyo, Japan). The fluorescence intensity (A.U.) was measured by software ImageJ.

### 3.9. Determination of Cell Survival Rate with X-Ray Radiation

Hep-2 cells were divided into groups with or without transfected with siRNA by Lipo or CSSA micelles, and further received 0 Gy (as control group) and 5 Gy radiation performed on accelerator linear (Clinac 23EX, Varian Medical Systems, Palo Alto, CA, USA). At 24 h, 48 h and 72 h after X-ray radiation, using MTT method, as described in 3.7 to determine the cell survival rate, which calculated with reference to the cells without X-ray radiation. All assays were carried out in triplicate.

### 3.10. Statistical Analysis

All data are reported as mean ± SD. Differences between groups were tested using the two-tailed student’s *t*-test (Prism 8.0). The differences with *p* < 0.05 were considered statistically significant.

## 4. Conclusions

In this work, we developed a chitosan-based glycolipid polymers chitosan oligosaccharide grafted stearic acid (CSSA). CSSA could self-aggregate above 75.02 ± 1.27 μg/mL in aqueous medium to form nano-sized micelles (144.7 ± 3.2 nm). As a cationic polymeric vector, CSSA could conjugate siRNA803 via electrostatic interaction with the selected proper N/P of 60. Comparing with the traditional commercial vector Lipo, CSSA exhibits significant lower cytotoxicity, better siRNA delivery ability. In consequence, the cell survival rates of Hep-2 transfected by CSSA/siRNA were significantly reduced comparing with either BR or Lipo/siRNA groups. As a promising polymeric vector for efficient siRNA transfection, CSSA conjugated siRNA delivery system CSSA/siRNA could enhance the radiosensitivity of laryngocarcinoma.

## Figures and Tables

**Table 1:** Characteristics of CSSA and CSSA/siRNA micelles.

Micelles	*d_n_* (nm)	PDI	Zeta Potential (mV)	SD (%)	CMC (μg/mL)
CSSA	144.7 ± 3.2	0.212 ± 0.031	39.7 ± 0.8	15.70 ± 1.02	75.02 ± 1.27
CSSA/siRNA	168.1 ± 2.9	0.136 ± 0.025	20.3 ± 0.5	-	-

The PDI, SD and CMC values represent the polydispersity index, degree of amino substitution and critical micelle concentration, respectively. CSSA/siRNA is prepared with N/P of 60. Data represent the mean ± standard deviation (n = 3).

## Data Availability

Not applicable.

## Sample Availability

Samples of the CSSA are available from the authors.
